# Precise gene models using long-read sequencing reveal a unique poly(A) signal in *Giardia lamblia*

**DOI:** 10.1261/rna.078793.121

**Published:** 2022-05

**Authors:** Danielle Y. Bilodeau, Ryan M. Sheridan, Balu Balan, Aaron R. Jex, Olivia S. Rissland

**Affiliations:** 1 Department of Biochemistry and Molecular Genetics, University of Colorado School of Medicine, Aurora, Colorado 80045, USA; 2 RNA Bioscience Initiative, University of Colorado School of Medicine, Aurora, Colorado 80045, USA; 3 Population Health and Immunity Division, The Walter and Eliza Hall Institute of Medical Research, Parkville, Melbourne, VIC 3052, Australia; 4 Faculty of Veterinary and Agricultural Sciences, The University of Melbourne, Parkville, VIC 3052, Australia

**Keywords:** 3′ UTR, *Giardia lamblia*, long-read sequencing, poly(A) site

## Abstract

During pre-mRNA processing, the poly(A) signal is recognized by a protein complex that ensures precise cleavage and polyadenylation of the nascent transcript. The location of this cleavage event establishes the length and sequence of the 3′ UTR of an mRNA, thus determining much of its post-transcriptional fate. Using long-read sequencing, we characterize the polyadenylation signal and related sequences surrounding *Giardia lamblia* cleavage sites for over 2600 genes. We find that *G. lamblia* uses an AGURAA poly(A) signal, which differs from the mammalian AAUAAA. We also describe how *G. lamblia* lacks common auxiliary elements found in other eukaryotes, along with the proteins that recognize them. Further, we identify 133 genes with evidence of alternative polyadenylation. These results suggest that despite pared-down cleavage and polyadenylation machinery, 3′ end formation still appears to be an important regulatory step for gene expression in *G. lamblia*.

## INTRODUCTION

Pre-mRNA processing is central to the proper expression and function of a gene. In eukaryotes, pre-mRNA processing involves capping, splicing, and cleavage and polyadenylation, which occur before export to the cytoplasm, and errors at any of these steps can have important consequences for gene expression. During cleavage and polyadenylation, the nascent RNA is cleaved at a precise location, which establishes the 3′ end of the mature transcript, and a poly(A) tail is added, which is required for downstream events in gene expression (Gallie 1991; Singh et al. 2015). In addition, some genes contain more than one cleavage site, resulting in isoforms with different 3′ UTRs and often different post-transcriptional fates (Tian et al. 2005; Sandberg et al. 2008; Mayr and Bartel 2009). Alternative polyadenylation (APA) is widespread in many eukaryotic species, including *S. cerevisiae*, *S. pombe* and plants, and more than half of human and mouse genes have multiple mRNA cleavage sites (Tian et al. 2005; Lu et al. 2006; Xing and Li 2011; Hoque et al. 2013; Liu et al. 2017; Moqtaderi et al. 2018). Inappropriate cleavage and polyadenylation can have severe, widespread consequences for gene expression and is associated with cancer and lethality (Whitelaw and Proudfoot 1986; Morris et al. 2012; Nourse et al. 2020), highlighting the central importance of this processing step.

Cleavage and polyadenylation is a complex, highly coordinated step that must be highly specific and sensitive. In humans, this process involves 20 core proteins and several *cis*-acting elements in the mRNA (Kumar et al. 2019). The main sequence element that directs cleavage is the polyadenylation signal [known as the poly(A) signal], which is an AAUAAA hexamer in metazoans (Proudfoot and Brownlee 1976; Beaudoing 2000). This hexamer and variants, such as AUUAAA, are in turn recognized by a multiprotein complex known as the cleavage and polyadenylation specificity factor (or CPSF), which is composed of CPSF160, CPSF30, WDR33, CSPF73, CPSF100, Symplekin, and Fip1 (Chan et al. 2011; Schönemann et al. 2014). Of these proteins, two (CSPF30 and WDR33) recognize and bind the poly(A) signal and, through other members of the complex, initiate cleavage (Chan et al. 2014; Clerici et al. 2018; Sun et al. 2018). Although not as clearly defined as in metazoans, A-rich motifs in budding yeast (such as AAGAA) play an analogous role as poly(A) signals (Gross and Moore 2001; Hill et al. 2019; Kumar et al. 2019).

In multiple species, the AAUAAA hexamer is insufficient to direct cleavage, and additional auxiliary sequences within the nascent transcript strengthen the poly(A) signal to promote accurate cleavage and polyadenylation (Sheets et al. 1990; Birse 1997). There are two major auxiliary elements in metazoans: upstream U-rich motifs and downstream U- and GU-rich motifs. The most highly enriched U-rich motif is a UGUA tetramer recognized by proteins in the Cleavage factor Im (CFIm) family (Brown and Gilmartin 2003; Venkataraman 2005). U- and GU-rich sequences downstream from the cleavage site are recognized by Cleavage stimulation factor proteins (CstF) that also help to strengthen the poly(A) signal and direct the endonuclease CPSF73 for cleavage of the nascent RNA (Takagaki and Manley 1997; Hu et al. 2005; Mandel et al. 2006; Sullivan et al. 2009). In yeast, similar auxiliary elements also help define cleavage sites (Dichtl 2002; Baejen et al. 2014).

Despite our deep knowledge of cleavage and polyadenylation in metazoans and yeast, less is known about the sequences and complexes involved in this process for other eukaryotes. There are over 200,000 species of protists, but we know poly(A) signals for only a handful. For instance, *Entamoeba histolytica,* which is found in the Amorphea supergroup alongside humans and yeast, uses an AAWUDA poly(A) signal (where W can be U or A, and D is any nucleotide but C), reminiscent of the metazoan signal (Hon et al. 2013). Similarly, there has been extensive research on pre-mRNA processing in kinetoplastids, such as *Trypanosoma* and *Leishmania* (Clayton and Michaeli 2011; Li and Du 2014). Unlike all other eukaryotes, kinetoplastids transcribe genes as polycistronic mRNAs, which are then cleaved to generate individual transcripts (Campbell et al. 2003; Clayton 2019). Although trypanosomes contain most of the conserved eukaryotic cleavage and polyadenylation proteins, the cleavage site is established by the trans-splicing of the upstream gene and is not dependent on a specific motif (Hendriks et al. 2003; Clayton 2013, 2019; Koch et al. 2016). For other protists, the mechanism of cleavage and polyadenylation is less well understood. For instance, a sequencing analysis of *Sarcocystis neurona, Neospora caninum,* and *Toxoplasma gondii* was unable to detect a poly(A) signal, although at least in *S. neurona*, there appears to be alternative polyadenylation during development (Stevens et al. 2018). *Plasmodium falciparum*, another apicomplexan, also seems to lack a clearly defined poly(A) signal (Oguariri et al. 2006; Siegel et al. 2014). Thus, a substantial amount of eukaryotic diversity remains unexplored for pre-mRNA cleavage and polyadenylation.

One protist that has attracted our interest is *Giardia lamblia*. A human parasite, *G. lamblia* is the causative agent of giardiasis, one of the most common intestinal diseases worldwide (Ankarklev et al. 2010). The *Giardia* clade encompasses multiple species that colonize the intestines of a variety of animals. Within the *Giardia* clade, *G. lamblia* is the sole species with the advantage of growing easily in axenic culture (Meyer 1976; Keister 1983). Although the exact placement of *Giardia* species on the eukaryotic tree of life is an ongoing area of investigation (Cacciò and Ryan 2008; Monis et al. 2009), it is generally understood to have branched off from traditional model systems, such as *Saccharomyces cerevisiae* and *Drosophila melanogaster*, relatively early and has been evolving independently for a long time. Recent phylogenetic analyses place *Giardia* within the Metamonada supergroup alongside other anaerobic protists like *Trichomonas*, although the term “Excavata” has also been used to describe this supergroup (Burki et al. 2020). Moreover, due to its ease of growth in the laboratory and its divergence from traditional model systems, *G. lamblia* presents an opportunity for studying highly conserved processes to see how these compare to what has been previously established.

From the perspective of gene regulation, *G. lamblia* differs from model organisms in several important ways. First, previous work has suggested that the 3′ UTRs of *G. lamblia* are unusually short, with a median of less than 100 nt (Franzén et al. 2013). This observation has raised fundamental questions about the potential for 3′ UTR-mediated post-transcriptional regulation in this organism. Second, consistent with short UTR regions, the genome of *G. lamblia* is generally very compact such that only eight genes contain introns and five undergo trans-splicing, while the number of protein-coding genes is between 5000 to 9000, depending on genome annotation (Xu et al. 2020a). Third, *G. lamblia* has streamlined machinery for transcription (Best 2004; Morrison et al. 2007), splicing (Nixon et al. 2002; Iyer et al. 2019), and translation (Li and Wang 2004; Eiler et al. 2020), and lacks many protein components that are essential for viability in most other eukaryotes, such as the translation initiation factor eIF4G (Li and Wang 2004; Morrison et al. 2007). Finally, *G. lamblia* exists in two forms, a dormant and hardy cyst and an infectious trophozoite, making it a potential model system to investigate how cell state and developmental transitions affect gene expression. However, despite growing interest in *G. lamblia*, fundamental aspects of pre-mRNA processing, including the identity of its poly(A) signal, remain unknown.

To provide an initial genome-wide characterization of *G. lamblia* 3′ end processing, we generated high-quality *G. lamblia* 3′ UTR annotations using two orthogonal high-throughput sequencing methods. Using these data, we identified the *G. lamblia* poly(A) signal as AGURAA (where R indicates a purine). Unlike yeast, *G. lamblia* uses a specific hexamer as its poly(A) signal. However, this sequence differs from that of metazoans at the second position, using a G rather than an A. This unusual poly(A) signal has shaped the *G. lamblia* genome, with the hexamer depleted in coding regions and yet, at times, also overlapping with stop codons to give extremely short 3′ UTRs. We found little evidence that known auxiliary sequences play a role in cleavage and polyadenylation, and many of the proteins that would recognize auxiliary sequences seem to also be absent. Together, our results suggest that *G. lamblia* has pared-down pre-mRNA processing machinery or that the sequences and complexes have diverged to the point where they are difficult to identify. Finally, we identified 133 genes with more than one cleavage site. These results increase the number of alternative polyadenylation events in *G. lamblia* by over 60-fold (Que et al. 1996; Mok et al. 2005). Our results suggest that, despite simplified cleavage and polyadenylation machinery, 3′ end formation is an important and as-yet underappreciated, mechanism for regulating gene expression in *G. lamblia*.

## RESULTS

### Characterization of *G. lamblia* mRNA 3′ ends at nucleotide resolution

To annotate *G. lamblia* 3′ UTRs, we began with a commercially available 3′-end sequencing method (QuantSeq), which uses an oligo(dT) primer to sequence 3′ ends of polyadenylated RNA with nucleotide resolution. We generated two replicate libraries using trophozoite RNA. Putative cleavage sites were defined by identifying the positions of read peaks downstream from annotated coding regions. Peaks that were within 10 nt of each other were merged into a single site. We filtered the sites to only include those where the predicted 3′ UTR showed at least 90% overlap between biological replicates. In some cases, these libraries led to multiple putative cleavage sites per gene, some of which were found tens of thousands of nucleotides away from the nearest open reading frame.

Given the known artifacts of this method (such as internal priming (Nam et al. 2002; Adiconis et al. 2013]) and the challenge of working with a relatively poorly annotated genome, we next used an orthogonal method to validate cleavage sites predicted by the 3′-end seq libraries. We directly sequenced *G. lamblia* RNA in duplicate using Oxford nanopore technology (ONT) and obtained 1.1 million total reads. ONT sequencing yielded long reads (average length: 940 bp) that enabled us to unambiguously develop precise gene models and thus enhance the transcriptomic map of *G. lamblia*.

**FIGURE 1.**
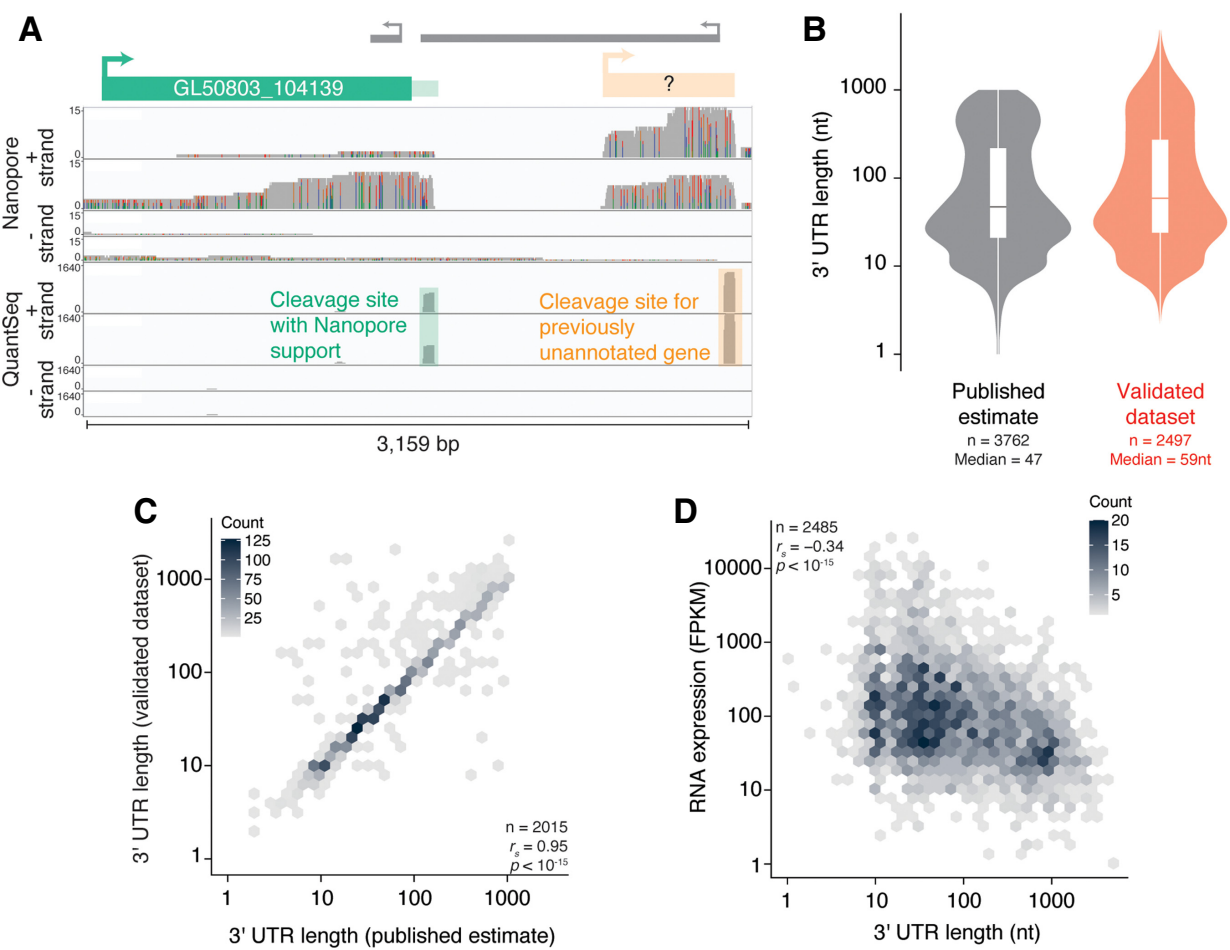
Characterization of *G. lamblia* 3′ ends at nucleotide resolution. (*A*) Genome browser image looking at the 3′ end of GL50803_104139 and displaying coverage of ONT libraries (*top*) and 3′-end libraries (*bottom*). Of the two cleavage sites predicted by the 3′-end libraries, one is supported by the ONT libraries (green box), while the other appears to belong to a previously unannotated transcript (orange box). (*B*) Distribution of 3′ UTR lengths in previously published work ((Franzén et al. 2013), *left*) and this study (*right*). (*C*) Hexagonal heatmap comparing published estimates of 3′ UTR lengths (*x*-axis) and the new data set from this study (*y*-axis). (*D*) 3′ UTR length is negatively correlated with expression. Shown is a hexagonal heatmap comparing 3′ UTR length (this study) and mRNA expression in Fragments Per Kilobase of transcript per Million mapped reads (FPKM, from accession number GSE158187).

We used two criteria for ONT read inclusion: reads were required to (1) have a poly(A) tail of at least 30 nt (suggesting that they were derived from mature transcripts, see below) and (2) extend into the open reading frame of the nearest gene (suggesting that they were genuine transcripts from that gene). To validate a cleavage site, we required that it was included in our QuantSeq data set and had at least one read in either of the two replicate ONT libraries. This method allowed us to remove cleavage sites resulting from internal priming as well as misassigned sites, such as those that belonged to previously unannotated genes (Fig. 1A). With this combined approach, we were able to identify 2764 cleavage sites across 2630 genes (which we will refer to as “validated cleavage sites,” Supplemental Fig. S1A).

To validate our results, we first compared them to the 3′ UTR lengths that had been previously determined experimentally. For instance, cyst wall protein 1 (CWP1) has been described as having a 36-nt 3′ UTR (Hehl et al. 2000), and our measurement gave 37 nt (Supplemental Table S1). Likewise, we found that NADP-specific glutamate dehydrogenase (GDH) has a 22-nt long 3′ UTR (Supplemental Fig. S1B; Supplemental Table S1), consistent with previous predictions (Yee and Dennis 1992). Thus, by using a combination of 3′-end seq and long-read sequencing, we generated a high-confidence data set of validated cleavage sites for thousands of *G. lamblia* genes.

We next compared our annotations with those previously predicted on a genome-wide scale (Franzén et al. 2013). The 3′ UTR lengths generated by our approach had a median of 59 nt and a similar distribution to previous predictions (Fig. 1B). Although these previous estimates and our own annotations were highly correlated (Spearman *r* [*r*_*s*_] = 0.95, *P* <10^−15^, Fig. 1C), for 693 genes our experimentally determined 3′ UTRs were longer than the previous predictions, highlighting the power of our approach. We also observed a significant negative correlation between 3′ UTR length and mRNA expression (Fig. 1D, *r_s_* = −0.34, *P* < 10^−15^), as has been observed in other organisms (Mayr 2017). This result raises the possibility that 3′ UTRs, despite their short length, may carry sufficient regulatory potential to modulate mRNA stability, although the associated mechanisms are unknown.

### ONT libraries characterize *G. lamblia* poly(A) tails for the first time

**FIGURE 2.**
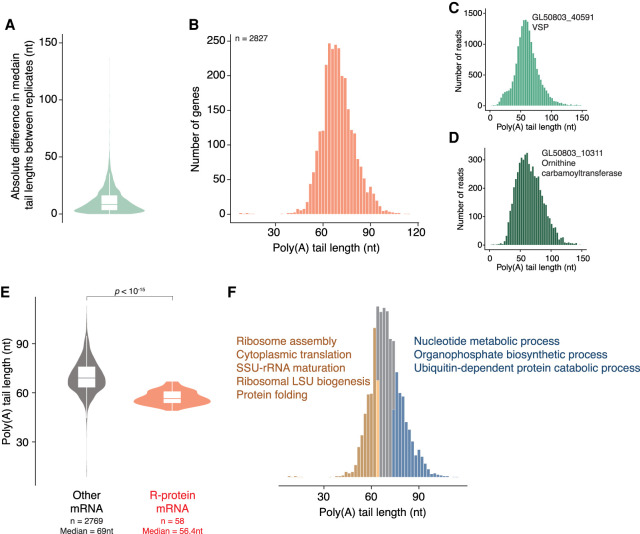
Poly(A)-tail measurements provide new insights. (*A*) Violin plot showing the absolute difference in poly(A)-tail measurements between ONT replicates. (*B*) Distribution of median poly(A)-tail length across both ONT replicates. Only mRNAs with a combined minimum of 10 reads are included. Median is 69 nt. (*C*) Distribution of poly(A)-tail lengths for reads aligning to GL50803_40591. (*D*) As in *C* but for GL50803_10311. (*E*) Comparison of poly(A)-tail length between mRNAs encoding ribosomal proteins (median 56.4 nt) and all other mRNAs (median 69.0 nt). Only genes with a minimum of 10 ONT reads were selected for this analysis. (*F*) GEO enrichment terms for genes with short (orange) or long (blue) poly(A) tails. Only genes with a minimum of 10 ONT reads were selected for this analysis.

The long-read libraries generated with ONT also allowed us to directly measure poly(A) tails in *G. lamblia* (Supplemental Fig. S2A). This aspect of RNA biology has been unexplored in *G. lamblia*, despite it being critical for understanding post-transcriptional regulation and for determining the extent to which standard methods [such as oligo(dT) selection] are appropriate for use in this organism. To examine the reproducibility of our measurements, we first compared the median tail lengths between the two ONT replicates, restricting our analysis to mRNAs with at least ten reads in both replicates. The tail lengths were significantly correlated (Supplemental Fig. S2B, Pearson's *r* = 0.45, *P* < 10^−15^), and, even more encouragingly, the median absolute difference in measured tail length between replicates was 8 nt, indicating that our tail length measurements were reproducible (Fig. 2A).

To maximize both the resolution and reliability of our results, we next focused on those mRNAs with at least ten reads across both data sets for subsequent analyses (Supplemental Table S2). The median tail length across these transcripts was 69 nt, with 80% of mRNAs having tails between 58 and 83 nt, and 0.2% having tails shorter than 30 nt (Fig. 2B). These lengths are similar to those in *Drosophila* and human cells, but substantially longer than those in *S. cerevisiae* (Chang et al. 2014; Subtelny et al. 2014; Krause et al. 2019; Workman et al. 2019; Yu et al. 2020). Interestingly, seven genes showed tails that were reproducibly shorter than 30 nt (Supplemental Fig. S2C–E; Supplemental Table S2). Of these, four encode ribosomal RNAs, indicating that in *G. lamblia* structured RNAs are oligoadenylated. In many other eukaryotes, oligo(A) tails are mediated by the TRAMP complex and enable processing and degradation by the nuclear exosome (LaCava et al. 2005). Our data suggest that a similar pathway likely operates in *Giardia.* From a practical perspective, these tail length measurements indicate that methods using oligo(dT) enrichment steps are suitable for *G. lamblia* and are unlikely to bias results.

In *C. elegans*, poly(A)-tail lengths show phasing at ∼30 nt intervals, consistent with the footprint of the poly(A) binding protein on the poly(A) tail of transcripts that are associated with one or multiple copies of poly(A) binding protein (Lima et al. 2017). We therefore examined the ten most highly expressed genes in our data set to ask whether we could observe something similar, but no phasing was observed. The overall distribution of reads remained constant when looking individually at highly expressed genes (Fig. 2C,D), and we also saw no evidence of phasing when looking across all genes (Supplemental Fig. S2A).

Previous work in yeast, humans, and other eukaryotes (Subtelny et al. 2014; Lima et al. 2017; Rissland 2017) has shown that mRNAs encoding ribosomal proteins (r-proteins) have some of the shortest poly(A) tails in the transcriptome, and we next asked whether this trend held in *G. lamblia*. As in other eukaryotes, r-protein mRNAs had significantly shorter poly(A) tails than those on other mRNAs (median 56.4 vs. 69 nt, respectively; Mann–Whitney *U*-test, *P* < 10^−15^; Fig. 2E). To ask what biological processes were associated with short or long poly(A) tails, we determined GO enrichment in the genes whose mRNAs were in bins for the 30% shortest or longest median tail length (Fig. 2F; Ashburner et al. 2000; The Gene Ontology Consortium et al. 2021). Although the poor annotation of the *G. lamblia* genome and the abundance of hypothetical proteins can make these types of analyses challenging, several processes such as nucleotide metabolism and organophosphate biosynthesis were enriched in genes whose mRNAs had long poly(A) tails, while those having short tails were enriched for several other processes, including ribosome assembly, cytoplasmic translation, rRNA maturation and protein folding. Taken together, these data indicate the underlying mechanisms leading to highly expressed mRNAs, like those encoding r-proteins, are conserved in *G. lamblia* despite its pared-down molecular machinery.

### *Giardia lamblia* uses an unusual poly(A) signal

**FIGURE 3.**
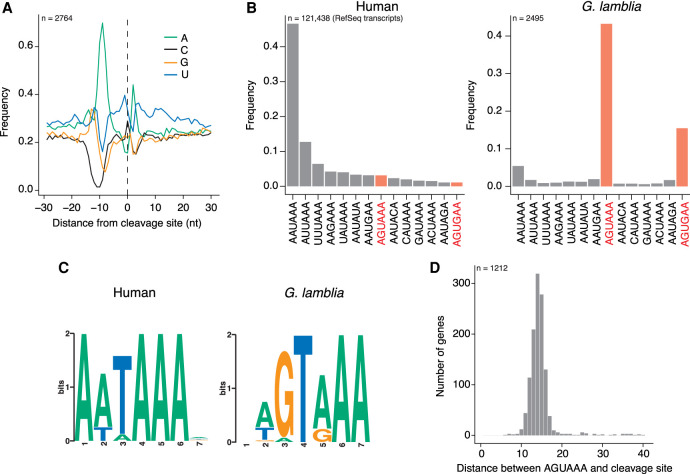
*G. lamblia* uses an unusual poly(A) signal. (*A*) Nucleotide frequency in the 60-nt window centered on all 2860 validated cleavage sites from this study. (*B*) Frequency of common poly(A) signals identified in studies of human transcripts (Beaudoing 2000). Sequences 30 nt upstream of cleavage sites from the human RefSeq annotations and validated *G. lamblia* sites from this study were used to search for common motifs. Plotted is the frequency of each signal in human (*left*) and *G. lamblia* (*right*). (*C*) MEME analysis of upstream sequences. The same sequences as in *B* were uploaded to the meme-suite, and a search was conducted for enriched hexamers. Shown is the top motif for human (*left*) and *G. lamblia* (*right*). (*D*) For all validated cleavage sites containing an AGUAAA motif in the last 40 nt of the mRNA, this bar graph shows the distance between the motif and the end of the read. Distances are counted from the first A of the motif.

From our list of validated cleavage sites, we next asked which poly(A) signals, if any, *G. lamblia* uses. As a first approach, we looked at the frequency of each nucleotide in a 60-nt window centered on the validated cleavage sites (Fig. 3A). We noticed A-richness approximately 10 nt upstream of the cleavage site, as well as a distinct A-peak directly downstream. There was also an enrichment of U nucleotides both up and downstream from this region, similar to that seen in other organisms (Supplemental Fig. S3A; Tian et al. 2005; Tian and Graber 2012). These results suggest that *G. lamblia* has sequence preferences for defining cleavage sites.

We next wanted to define the precise poly(A) signal used in *G. lamblia*. To do so, we focused on genes with only one validated cleavage site and counted the occurrences of hexameric motifs previously identified in humans (Beaudoing 2000). When we performed this analysis on human RefSeq transcript annotations, AAUAAA was the most abundant polyadenylation signal, as expected (Fig. 3B). In contrast, distinct but related motifs were the most highly enriched in our *G. lamblia* data set: AGUAAA and AGUGAA were found in 45% and 15% of genes, respectively. In contrast, AAUAAA was used more rarely and occurred in only 5% of genes.

As an independent approach, we searched for hexameric motifs occurring within the first 30 nt upstream of human and *G. lamblia* cleavage sites using the MEME package (Bailey et al. 2009). This unbiased approach confirmed the strong enrichment for the G nucleotide at position 2 of the *G. lamblia* poly(A) signal and the strong preference for a purine at position 4 (Fig. 3C). Our identified poly(A) signal is also consistent with early studies of individual *G. lamblia* genes that suggested an AGURAA motif as the polyadenylation signal (Peattie et al. 1989; Yee and Dennis 1992; Que et al. 1996)—an observation we have now confirmed on a genome-wide scale.

Interestingly, although metazoan poly(A) signals are usually found 10 to 30 nt upstream of the cleavage site (Supplemental Fig. S3B; Kumar et al. 2019), *G. lamblia* signals tended to be closer to the cleavage site (Fig. 3D). In over 90% of genes with an AGUAAA signal, the motif was <20 nt from the cleavage site, and the most common distance was 13–15 nt, an observation consistent with the general compactness of the *G. lamblia* genome.

### Implications of unusual poly(A) signal on the *G. lamblia* genome

**FIGURE 4.**
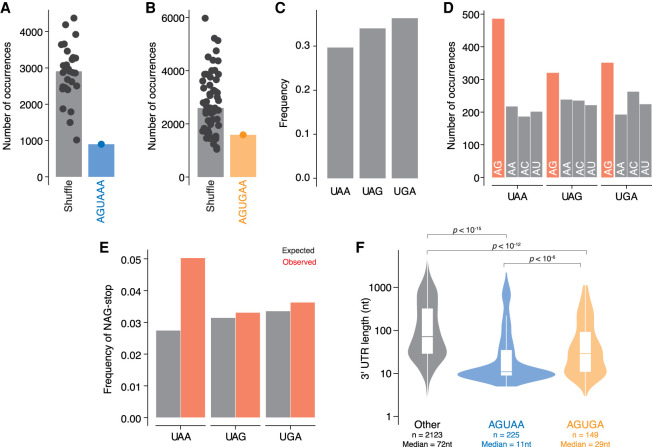
Implications of unusual poly(A) signal on *G. lamblia* open reading frames. (*A*) Open reading frames are depleted for *G. lamblia's* poly(A) signal. Open reading frame sequences were used to count the occurrence of AGUAAA vs all shuffled versions of the motif. (*B*) As in *A*, but with the AGUGAA poly(A) signal. (*C*) Frequency of stop codons across all annotated *G. lamblia* open reading frames. (*D*) Nucleotides preceding a stop are enriched for AG over other AN dinucleotides. For each stop codon, this bar graph shows how many were preceded by the different AN dinucleotide sequences. (*E*) As in *D*, but comparing expected versus observed frequencies. The expected frequency for each sequence context was calculated from the total frequency of each codon across all open reading frames. (*F*) Distribution of 3′ UTR lengths for genes where there is no overlap of poly(A) signal and stop codon (*left*), genes where there is an AG dinucleotide preceding a UAA stop codon (*middle*), and genes where there is an AG preceding a UGA stop codon (*right*).

We next wished to investigate how the unusual poly(A) signal has shaped the *G. lamblia* genome. First, given that AGUAAA and AGUGAA are poly(A) signals, we would expect them to be depleted in open reading frames as their presence could lead to premature cleavage. To test this prediction, we counted the occurrence of both motifs and compared them to the frequency of their shuffled sequences (e.g., AAUAGA). We found that AGUAAA is strongly depleted in open reading frames compared to the shuffled sequences, while the depletion of AGUGAA was more modest, consistent with the prediction that AGUAAA is the preferred signal (Fig. 4A,B).

We then investigated the relationship between poly(A) signals and stop codons. A recent study of *Giardia muris* reported that many genes have an overlap between these signals (Xu et al. 2020b), and genomic analysis of *Spironucleus salmonicida*, another diplomonad, has likewise indicated a strong “dual use” of poly(A) signals as stop codons (Xu et al. 2014). In the case of *S. salmonicida*, the stop codon (UGA) is predominantly used throughout the genome, overlapping with a predicted AGUGA poly(A) signal (Xu et al. 2014). Given the short length of 3′ UTRs in *G. lamblia*, we wondered whether this overlap of signals might also occur here. We first calculated the frequency of each stop codon across all open reading frames. We did not observe a strong preference for any stop codon, and UAA (which would allow for an AG–UAA motif) was the least abundant of the three stop codons (Fig. 4C). We next looked more closely at the nucleotides preceding the stop codon and asked whether there was a preference for AA, AU, AC, or AG. Of these, only an (N)AG sequence in front of the stop codon will allow for a dual AGUAAA or AGUGAA poly(A) signal/stop codon combination. Although there was no enrichment for the UAA stop codon itself, it was much more likely to be preceded by an NAG codon than the other codons. We also observed a preference for AG dinucleotides preceding UGA, and a more modest enrichment for UAG, which would not support a dual-use poly(A) signal/stop codon (Fig. 4D). In contrast, AA dinucleotides showed no such preference, providing an additional line of support that *G. lamblia* does not use the AAUAAA hexamer.

Two alternative models could explain the nucleotide bias in the codon preceding the stop codon: The first is that NAG–UAA and NAG–UGA represent genuine poly(A) signals, and the second is that their presence is simply a consequence of codon usage or amino acid preferences. To distinguish between these possibilities, we compared the expected and observed frequencies of NAG sequences preceding the stop codon. Consistent with NAG–UAA serving as a dual poly(A) signal/stop codon, this pair occurred more frequently than expected based on the frequencies of either alone. The same was not true for NAG–UGA (Fig. 4E). To investigate this issue further, we examined the 3′ UTR lengths of genes with the potential dual use AG–UAA or AG–UGA stop codons. Compared with other genes, 3′ UTR lengths were shorter for both AGUAA- and AGUGA-ending transcripts (*P* < 10^−15^ and *P* < 10^−12^, respectively; Fig. 4F). In the case of AGUAA, the median length was 11 nt, which is in the window of distances between genuine poly(A) signals and cleavage sites. These analyses indicate that NAG–URA sequences can act as genuine dual-use stop codons and poly(A) signals. In other words, in *G. lamblia,* stop codons have acquired the ability to also act as poly(A) signals for ∼15% of genes. This dual usage has not reached the levels predicted in *G. muris* and *S. salmonicida*, suggesting that this aspect of genome organization is evolving relatively rapidly within the diplomonad order.

### Eukaryotic auxiliary elements are poorly enriched around *G. lamblia* cleavage sites

We have an advanced understanding of the sequences and proteins involved in recognition of polyadenylation signals and auxiliary elements in other eukaryotes. In metazoans, there are three main complexes that recognize the polyadenylation signal, upstream U-rich motifs and downstream U- and GU-rich motifs: CPSF, CFIm and CstF complexes, respectively (Takagaki and Manley 1997; Brown and Gilmartin 2003; Kumar et al. 2019). However, it is completely unknown whether *G. lamblia* also makes use of auxiliary elements to define cleavage sites.

**FIGURE 5.**
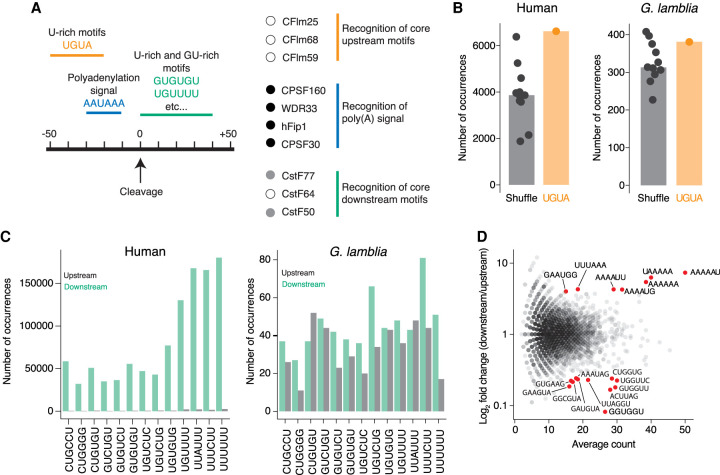
Conserved auxiliary elements are poorly enriched around *G. lamblia* cleavage sites. (*A*) Conserved pre-mRNA processing proteins and the sequences they recognize. The *left* panel shows the location and motifs of key sequences found around human cleavage sites. *Right* panel shows the human orthologs of core processing complexes for the recognition of poly(A) signals and surrounding sequences. Dots indicate whether an ortholog was readily identifiable in *G. lamblia* (black circle), whether ortholog identification was ambiguous (gray circle), or whether no orthologs were found (white circle). (*B*) The conserved UGUA motif is not enriched upstream of *G. lamblia* cleavage sites. Sequences 20 to 50 nt upstream of cleavage sites were used to count the frequency of UGUA or shuffled versions of the motif. Plotted is the number of times each motif was found in human (*left*) and *G. lamblia* (*right*) sequences. (*C*) GU-rich elements are not enriched downstream from *G. lamblia* cleavage sites. Sequences 40 nt up- and downstream from human and *G. lamblia* cleavage sites were used to count the occurrence of U- and GU-rich motifs enriched downstream from strong human cleavage sites (Hu et al. 2005). Plotted is the frequency of each motif upstream (gray) or downstream (green) of human (*left*) and *G. lamblia* (*right*) cleavage sites. (*D*) MA plot of enriched and depleted 6-mer sequences around polyadenylation signals. All single cleavage site genes from our data set that contain an AGUAAA were selected for this analysis. Sequences 50 nt upstream and downstream from the signal were used to search for all possible 6-nt motifs. Plotted is the average count of each motif versus its enrichment in downstream sequences. Red dots are motifs that showed at least a fourfold enrichment or depletion in downstream regions and with an average count of at least 15 occurrences.

To investigate whether these sequences were conserved in *G. lamblia*, we began by searching for orthologs to the associated proteins. Although we readily identified candidates for the CPSF complex [which recognizes the poly(A) signal], we found only low-confidence candidates for members of the CstF complex (which recognizes downstream U-rich motifs), and we were unable to identify orthologs for the CFlm proteins (which recognize upstream U-rich motifs and UGUA; Fig. 5A; Supplemental Table S3).

We next examined the sequences surrounding *G. lamblia* cleavage sites to investigate the extent to which the corresponding recognition sequences of these complexes were enriched. We interrogated sequences 20 to 50 nt upstream of the cleavage sites where the highly conserved UGUA motif is found in other eukaryotes (Brown and Gilmartin 2003; Millevoi and Vagner 2010). By counting the number of occurrences of UGUA as well as shuffled versions of the motif, we observed a strong preference for UGUA in the human genome, as expected. In contrast, we saw only a slight enrichment in *G. lamblia* (Fig. 5B). Consistent with this result, when we performed an unbiased motif search using MEME, no sequences were enriched in this region (data not shown). This poor sequence conservation, combined with our inability to identify any CFlm orthologs, suggest that upstream motifs either do not play a role in the processing of *G. lamblia* transcripts or are sufficiently divergent as to preclude identification.

Next, we searched for downstream auxiliary elements. In other organisms, these downstream elements lack a consensus motif, but rather are generally U-rich. Thus, we looked for hexamers that were enriched around strong poly(A) sites in human sequences (Hu et al. 2005). As expected, we found that U-rich sequences were highly enriched in regions downstream from cleavage sites in humans, but almost completely absent upstream. In contrast, in *G. lamblia* the sequences were equally present on either side of cleavage sites (Fig. 5C), which suggests that *G. lamblia* does not use conserved downstream auxiliary elements. However, because we observed a strong U bias downstream from the cleavage site in metagene analyses (Fig. 3A), and the ambiguous presence of putative CstF orthologs raise the possibility that instead divergent *cis-*elements and proteins may help define genuine cleavage sites, we turned to an unbiased approach to look for enriched motifs. For each gene containing a single cleavage site and an AGUAAA poly(A) signal, we searched for all possible 6-nt motifs in the 50 nt upstream and downstream from the signal. We found an enrichment for A-rich and AU-rich motifs in the downstream regions, and a depletion of more canonical GU-rich motifs (Fig. 5D). These results support our observation that any sequences that may help strengthen poly(A) signals in *G. lamblia* have diverged substantially from those found in classical model eukaryotes.

### Evidence of alternative polyadenylation in *G. lamblia*

**FIGURE 6.**
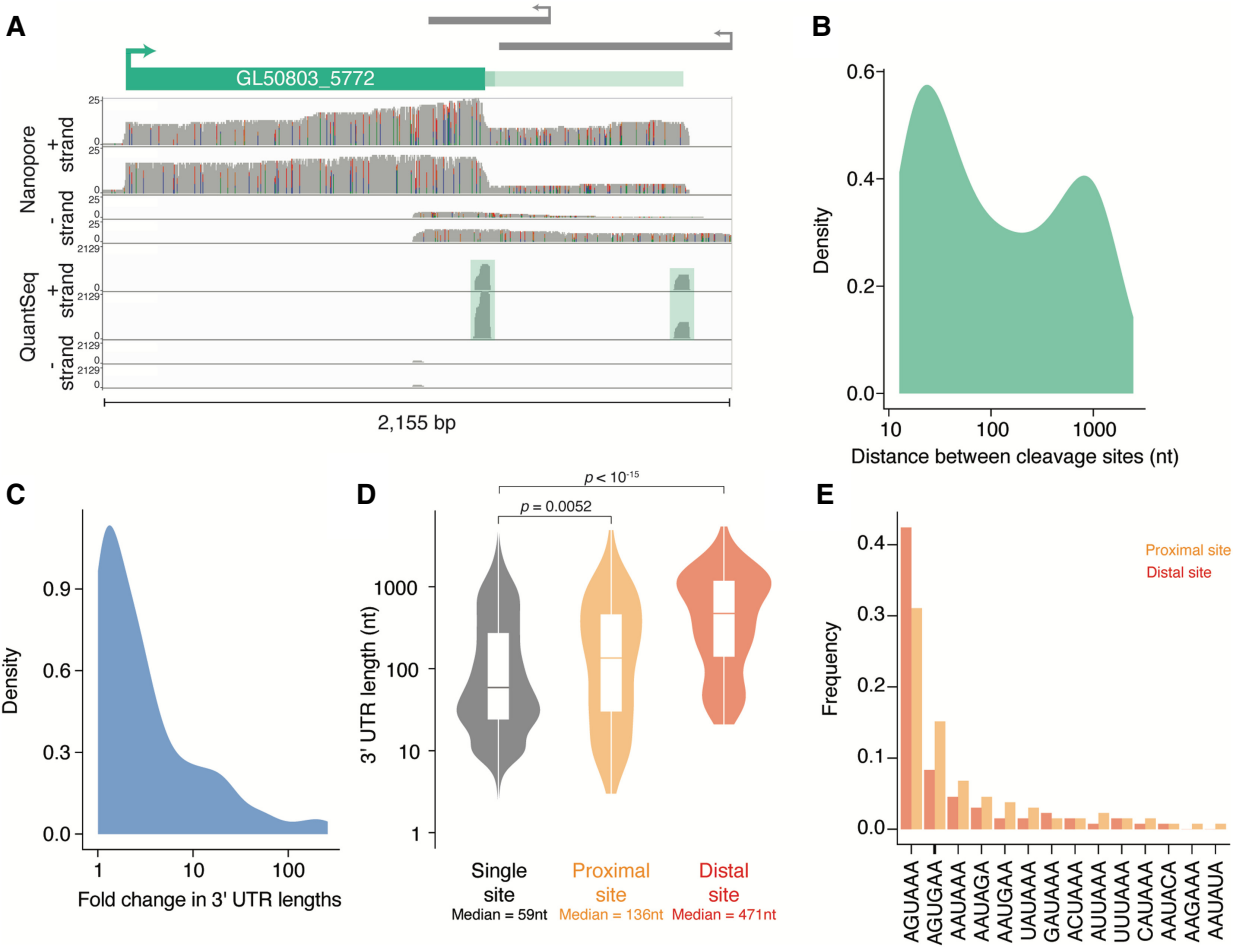
Evidence of alternative polyadenylation in *G. lamblia*. (*A*) Genome browser image looking at the 3′ end of GL50803_5772 and displaying coverage of ONT libraries (*top*) and 3′-end libraries (*bottom*). Both methods support the presence of two distinct cleavage sites for the gene. (*B*) Density plot showing the distribution of lengths between proximal and distal cleavage sites for the genes that have more than one cleavage site. The median is 81 nt. (*C*) Density plot showing the fold change in 3′ UTR length between distal and proximal cleavage sites. Median is a 2.18-fold change. (*D*) Distribution of 3′ UTR lengths for genes with a single cleavage site (*left*), the proximal sites for APA genes (*middle*), and the distal sites (*right*). (*E*) Poly(A) signal usage in APA genes. Sequences 30 nt upstream of proximal and distal cleavage sites were used to search for the motifs described in Figure 2B. Plotted is the frequency of each motif across proximal (orange) and distal (red) cleavage sites.

There are two previously described examples of alternative polyadenylation in the *G. lamblia* literature (Que et al. 1996; Mok et al. 2005; Einarsson et al. 2016), and so alternative polyadenylation has not been believed to be widespread. However, as mentioned above, when annotating cleavage sites, we unexpectedly found 133 genes showing evidence of alternative polyadenylation (Fig. 6A; Supplemental Table S1), suggesting that alternative polyadenylation may be more common in *G. lamblia* than previously suspected (Supplemental Fig. S4A).

The majority of these alternative cleavage sites were within 100 nt of each other, although for 20 genes the distal cleavage site was over 1000 nt downstream from the proximal one (Fig. 6B). Nonetheless, given the short length of 3′ UTRs in *G. lamblia*, in 53% of cases, usage of the distal site more than doubled the amount of regulatory sequence (Fig. 6C; Supplemental Fig. S4B). Interestingly, even usage of the proximal site resulted in longer 3′ UTRs than in the rest of the transcriptome (Fig. 6D: 136 nt vs. 59 nt, *P* = 0.0052). In humans, proximal sites often use “weaker” poly(A) signals than distal sites (Legendre and Gautheret 2003; Hu et al. 2005), and so we looked at poly(A) signals for these examples in *G. lamblia*. We found that distal cleavage sites are more likely to use AGUAAA and that proximal sites have a higher frequency of alternate signals such as AGUGAA, which is consistent with a preference for AGUAAA over AGUGAA in the transcriptome (Fig. 6E).

The presence of alternative poly(A) sites, as well as the generally longer 3′ UTR lengths observed, suggested that the regulation of this subset of genes may be biologically important. We observed a slight difference in overall expression between genes that had a single or multiple cleavage sites (median FPKM: 86 and 59.2, respectively; *P* = 0.00024; Supplemental Fig. S4C), although there was no difference in poly(A)-tail lengths (*P* = 0.39; Supplemental Fig. S4D). We also performed a gene ontology enrichment analysis, but no significant processes were enriched in genes undergoing alternative polyadenylation. We suspect that this result may be because more than 50% of genes are uncharacterized in *G. lamblia*, which limits the power of these approaches. Indeed, 12 of the alternative polyadenylation genes are described as “putative,” and 72 encode hypothetical proteins or unspecified products. Nonetheless, two ribosomal protein genes (*S4* and *S28*), as well as nine predicted kinases use alternative polyadenylation (Supplemental Table S1), raising the intriguing possibility that alternative polyadenylation may be important for the *G. lamblia* life cycle.

## DISCUSSION

Here, we empirically annotated the 3′ UTRs, for 2630 expressed genes in *G. lamblia* using a combination of 3′-end short- and long-read sequencing. According to our RNA-seq data (Eiler et al. 2020), 6616 of the 9700 predicted coding genes in the genome annotation used for this study are expressed at an FPKM of 10 or higher. This indicates that we have annotated about 40% of the expressed transcriptome. Although one barrier to annotating the rest of the genome is low ONT sequencing depth (relative to short-read based sequencing) and the very low RNA expression of the remaining genes (average FPKM = 1.89), direct long-read RNA sequencing was nonetheless instrumental in overcoming some of the difficulties associated with the study of an organism whose genome remains relatively unannotated compared to traditional model systems. Critically, our use of ONT sequencing mitigated known issues with 3′ end short read sequences (Adiconis et al. 2013) and directly linked cleavage sites and open reading frames.

Our work confirms the early putative hypothesis for the *G. lamblia* poly(A) signal (Peattie et al. 1989; Yee and Dennis 1992; Que et al. 1996) and demonstrates that *G. lamblia* uses AGURAA on a genome-wide scale. Interestingly, the most frequent signal (AGUAAA) differs from the metazoan AAUAAA motif by only a single nucleotide, using a G at position 2 rather than an A—but the two most common *G. lamblia* signals (AGURAA) are used only rarely in metazoans (Hu et al. 2005). An interesting future question is how this divergent sequence is recognized. In metazoans, the poly(A) signal is recognized by CPSF30 and WDR33 (Chan et al. 2014; Casañal et al. 2017; Clerici et al. 2018). We were able to identify putative orthologs to these key players, but orthologs for supporting proteins such as CPSF-100 and Symplekin remain to be found (Supplemental Table S3). The predicted CPSF30 ortholog in *G. lamblia* is similar to the human protein but contains four zinc finger (ZF) motifs instead of five, corresponding to motifs 2–5 in human CPSF30. Binding between CPSF30 and the AAUAAA motif is mediated by ZF2 and ZF3, suggesting that the core elements of poly(A) signal recognition are likely conserved in *G. lamblia* (Barabino et al. 2000; Schönemann et al. 2014; Kumar et al. 2019). Furthermore, the highly conserved residues on CPSF30 that are critical for recognition of the motif appear to be conserved and do not offer immediate insight into why *G. lamblia* uses a different signal. Identifying the appropriate orthologs and their sequence, structure, and biochemical preferences will be an important next step for understanding the basis of the unique *G. lamblia* poly(A) signal and its evolution.

Although starting with conserved eukaryotic sequences proved to be a good strategy when looking for polyadenylation signals, it was not the case for auxiliary elements. We were unable to find evidence of enrichment for any of the most common metazoan sequences that are found up or downstream from cleavage sites. It is therefore likely that any motifs outside the poly(A) signal used by *G. lamblia* to direct 3′-end processing have diverged significantly from those found in other eukaryotes, and their identification will likely require additional functional studies.

Finally, an unexpected finding from our study of 3′ UTRs is that 133 genes use alternative polyadenylation. Previous reports had identified only two cases (Que et al. 1996; Mok et al. 2005), a result that had led to a view that alternative polyadenylation was as rare as splicing in *G. lamblia.* Our results demonstrate that, contrary to this model, alternative polyadenylation is a more generally used mechanism, adding to the regulatory layers used by *G. lamblia*. Indeed, our results raise more intriguing questions about how cleavage and polyadenylation is regulated. For instance, how do these different 3′ UTR isoforms affect transcript stability and translation? Why do some genes use alternative polyadenylation and not others? Previous reports have suggested that encystation impacts gene expression as well as cleavage and polyadenylation of individual genes (Que et al. 1996; Mok et al. 2005; Einarsson et al. 2016). An intriguing possibility is that alternative polyadenylation may be especially important during this process or in the cyst itself (which is transcriptionally silent), and it will be exciting to explore this and other questions in the future.

## MATERIALS AND METHODS

### Trophozoite culture and RNA extraction

*Giardia lamblia* trophozoites (assemblage A, strain WB clone C6) were grown in modified TYS-33 media as per standard protocols (Keister 1983). Cells were harvested by placing culture tubes on ice for 10 minutes, then spun down for 5 minutes at 800x g at 4°C. Cell pellets were washed twice in 1xPBS. RNA was extracted from trophozoite pellets with hot acid phenol as previously described (Collart and Oliviero 1993).

### RNA sequencing and analysis

Previously generated RNA-seq libraries used in this study are available from the GEO (GSE158187). 3′-end libraries were generated with the QuantSeq 3′ mRNA-seq Library REV kit from Lexogen (catalog #016) according to the manufacturer's protocol. Libraries were sequenced at the Genomics and Microarray Shared Resource at the University of Colorado Denver Cancer Center. All sequencing data generated in this study are available from the GEO, accession number GSE168675.

Nanopore libraries were prepared according to the direct RNA sequencing protocol from ONT (SQK-RNA002). Because the lengths of poly(A) tails were unknown when we initiated this study, total RNA was used in place of oligo(dT)-selected RNA. Libraries were sequenced on a FLO-MIN106 flow cell and minION sequencing device. Base-calling was completed by the MinKNOW software (Nanopore) on default settings.

Adaptors were trimmed from 3′-end reads using Cutadapt v2.3. RNA-seq and QuantSeq libraries were aligned using STAR 2.5.2a (Dobin et al. 2013). Nanopore libraries were aligned with minimap2 version 2.17-r974-dirty (Li 2018). All libraries were mapped to the *Giardia lamblia* WBC6 genome version 50 downloaded from the GiardiaDB website on February 8, 2021 (https://giardiadb.org). Poly(A)-tail lengths from the Nanopore libraries were measured using Nanopolish version 0.11.1 (Loman et al. 2015). Mapped nanopore reads were assigned to their corresponding gene using featureCounts version 2.0.0 (Liao et al. 2014).

### Identification and validation of cleavage sites

3′ UTRs were annotated by first identifying poly(A) sites. Poly(A) sites were mapped by identifying peaks of poly(A) reads that aligned downstream from coding regions but did not overlap the following gene. Potential poly(A) sites were filtered to only include those that have at least ten reads. Sites that were within 10 nt of each other were combined into a single peak with coordinates representing the center point between the sites.

For each putative cleavage site, a list of coordinates was generated that went 10 nt up- and downstream from the site. For each gene, ONT reads with 3′ ends that ended within the corresponding window were selected. Reads were then further filtered to keep only those that contained a poly(A) tail of at least 30 nt and for which the 5′ end of the read fell within the open reading frame of the associated gene. Sites with at least one read from either replicate of the ONT libraries that satisfied all conditions were kept as validated sites. Analyses and plotting were performed in R version 4.0.3 and Python version 3.8.3 from in-house scripts. All genome browser images were generated with IGV version 2.8.10.

### Unbiased motif analysis

Motif-based sequence analysis was done using the MEME suite software at https://meme-suite.org (Bailey et al. 2009). We searched for a maximum of three motifs on the given strand only with minimum and maximum motif lengths of 6 and 50 nt, respectively.

### Ortholog identification

Human protein sequences were used to search for orthologs in *G. lamblia* by BLAST search. Where it was difficult to identify the most likely ortholog among the search results, the yeast protein sequence was used for a complementary search. Searches were conducted on https://giardiadb.org.

For CPSF160 and WDR33, human proteins containing similar domains were used to perform a multiple sequence alignment, which was then used to generate a hidden Markov model. We then initiated a search across the *G. lamblia* proteome in search of proteins that have a similar domain and sequence.

## SUPPLEMENTAL MATERIAL

Supplemental material is available for this article.

## Supplementary Material

Supplemental Material

## Figures and Tables

**Danielle Bilodeau:**
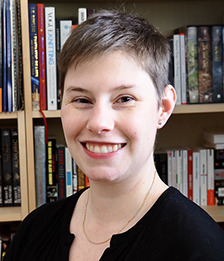


## MEET THE FIRST AUTHOR

***Meet the First Author(s)* is a new editorial feature within *RNA*, in which the first author(s) of research-based papers in each issue have the opportunity to introduce themselves and their work to readers of *RNA* and the RNA research community. Danielle Bilodeau is the first author of this paper, “Precise gene models using long-read sequencing reveal a unique poly(A) signal in *Giardia lamblia*.” Danielle did this work as a graduate student in the Rissland laboratory, first at the University of Toronto and then at the University of Colorado School of Medicine, where she studied RNA regulation in *Giardia lamblia* with a focus on 3′-UTR regulation and translation. Danielle is currently a post-doctoral researcher at Université Sainte-Anne in Canada where she is using genetic analysis to study reproductive strategies in a species of diving duck.**

**What are the major results described in your paper and how do they impact this branch of the field?**

Our work sheds light on some of the central aspects of RNA biology in *Giardia lamblia* and is the first to use long-read sequencing to study RNA in this organism. We provide a new high-resolution annotation of 3′-UTRs and evidence for over 100 genes that undergo alternative polyadenylation. Our study also identifies the poly(A) signal AGURAA as being present in over 60% of genes in our data set and explores how this uncommon signal has shaped the *G. lamblia* genome. By adding these important insights to the field's understanding of *G. lamblia* biology, we are providing fundamental resources for the continued study of an important human pathogen.

**What led you to study RNA or this aspect of RNA science?**

I didn't know I wanted to study RNA until I joined the Rissland laboratory and started to learn about all the wonderful aspects of RNA biology I had never imagined. I became especially curious about the regulation of RNA decay and what makes some transcripts more stable than others. When I came across *Giardia*, the fact that so many basic aspects of RNA regulation were still relatively unexplored got me excited about the potential for new and unexpected discoveries, and this curiosity became the foundation for my thesis work.

**During the course of these experiments, were there any surprising results or particular difficulties that altered your thinking and subsequent focus?**

When we started analyzing 3′-UTR lengths, we thought the large number of genes with more than one cleavage site might be sequencing artifacts. The possibility of alternative polyadenylation had been suggested before by other *Giardia* studies, but only in very unique cases. However, as we incorporated long-read sequencing and continued to study our data, we were surprised and excited to find that many genes do in fact undergo alternative polyadenylation, something we had never expected to find on such a scale.

**What are some of the landmark moments that provoked your interest in science or your development as a scientist?**

My love for science started when I was about 15 years old and learned about DNA replication in my biology class. The elegance and simplicity of it really fired my imagination and made me want to learn more. When it came time for my undergraduate studies, I was very fortunate to have access to a university close to home and enthusiastic professors who kept my curiosity alive and strong. In graduate school, joining the Rissland laboratory gave me the opportunity to learn the importance of doing the right experiment and the benefits of following the science even, and especially when, it goes in unexpected directions. My graduate school mentor played a huge role in shaping the scientist that I am today, and my experiences during my PhD taught me a great deal about what it means to do good science. Over the past couple years, the global pandemic has highlighted for me the importance of being able to explain complex notions to people who don't have a background in science, and how easy it is to be misled by ideas even when they lack convincing evidence.

**What are your subsequent near- or long-term career plans?**

In the short-term I am bringing my molecular biology expertise to ecology research and teaching an introductory genetics course to undergraduate students who will hopefully be left with a lasting fascination for nucleic acids. In the future, I hope to continue teaching in one form or another, as I've found sharing my passion for science and helping others understand the world around them to be the best part of being a scientist.
