# Supplementary material for: Precise gene models using long-read sequencing reveal a unique poly(A) signal in Giardia lamblia
Source: RNA. 2022 May;28(5):668–82. doi: 10.1261/rna.078793.121 (PMC9014877; doi:10.1261/rna.078793.121)
Supplement: Supplemental Material [file supp_28_5_668__DC1.html]

Precise gene models using long-read sequencing reveal a unique poly(A) signal in Giardia lamblia — Supplemental Material 

# Precise gene models using long-read sequencing reveal a unique poly(A) signal in *Giardia lamblia*

## Supplemental Material

- Supplemental\_Figure\_Legends.docx
- Supplemental\_Figure\_S1.pdf
- Supplemental\_Figure\_S2.pdf
- Supplemental\_Figure\_S3.pdf
- Supplemental\_Figure\_S4.pdf
- Supplemental\_Table\_1\_UTRLengths.xlsx
- Supplemental\_Table\_2\_TailLengths.xlsx
- Supplemental\_Table\_3\_Orthlogs.xlsx
