# Supplementary material for: Precise gene models using long-read sequencing reveal a unique poly(A) signal in Giardia lamblia
Source: RNA. 2022 May;28(5):668–82. doi: 10.1261/rna.078793.121 (PMC9014877; doi:10.1261/rna.078793.121)
Supplement: Supplemental Material [file supp_078793.121_Supplemental_Figure_Legends.docx]

**SUPPLEMENTAL FIGURE LEGENDS**

**Supplemental figure 1. ONT sequencing allows for validation of cleavage sites.** (A) Table showing the number of genes and sites for each step in our pipeline. "QuantSeq overlap" represents the 3′ UTRs that show at least 90% overlap between biological replicates. (B) Genome browser image looking at the 3′ end of GL50803_21942 (NADP-specific glutamate dehydrogenase). 3′-end libraries and ONT libraries both show a cleavage site 22 nucleotides downstream from the stop codon, which is consistent with previous observations.

**Supplemental figure 2. Poly(A)-tail lengths in *G. lamblia*.** (A) Distribution of poly(A)-tail lengths in ONT libraries. Histogram shows the poly(A)-tail length of all reads that were assigned to a gene and for which a poly(A) tail was measured. Median is 62.1 nucleotides. (B) Hexagonal heatmap comparing poly(A)-tail measurements between ONT replicates. mRNAs with at least ten reads in both ONT datasets were selected and median poly(A)-tail lengths compared. (C-E) Genome browser images looking at GL50803_r0021 (28S ribosomal RNA), GL50803_r0019 (18S ribosomal RNA) and GL50803_r0020 (18S ribosomal RNA). Colored arrow illustrates the length and direction of the annotated gene. Grey arrows represent other genes present on the chromosome or contig being displayed. Shown are ONT reads on the positive (top) and negative (bottom) strands from two biological replicates.

**Supplemental figure 3. Cleavage and polyadenylation contexts in humans.** (A) Nucleotide frequency surrounding human cleavage sites. Sequences surrounding the end coordinates of all mRNAs in the RefSeq annotation were used to calculate nucleotide frequencies 30 nucleotides upstream and downstream of cleavage sites. (B) Distances between AAUAAA poly(A) signal and cleavage sites in human transcripts. mRNA sequences were downloaded from RefSeq annotations. All sequences with an AAUAAA in the last 40 nucleotides of the transcript were selected and the distance was calculated between the motif and the end of the mRNA. Distances are counted from the first A of the motif.

**Supplemental figure 4. Gene differences between proximal and distal cleavage site usage.** (A) The number of alternative polyadenylation events in *G. lamblia* is greater than previously thought. Bar plot shows the number of genes that were believed to contain more than one cleavage site based on previous literature, our 3′-end libraries alone (QuantSeq), and our validated list of cleavage sites (Nanopore). (B) Genome browser image showing a gene (GL50803_27887) with a short and long 3′ UTR measuring 86 and 984 nucleotides, respectively. (C) FPKM expression for genes with a single cleavage site (left) or multiple cleavage sites (right). Median values are 86 and 59.2, respectively. (D) Distribution of poly(A)-tail lengths for genes with a single cleavage site (left) or multiple cleavage sites (right). Median values are 69 and 68.2 nucleotides, respectively.

**Supplemental table 1. Validated 3′ UTR sites in *G. lamblia*.** Table of all validated 3′ UTR lengths generated in this study. Genes are in separate sheets according to the number of cleavage sites.

**Supplemental table 2. Poly(A)-tail lengths for *G. lamblia* transcripts.** Table of poly(A)-tail lengths for transcripts with at least ten reads in either ONT replicate. Listed are the number of reads in each replicate, median tail lengths and gene descriptions.

**Supplemental table 3. Predicted orthologs for cleavage and polyadenylation proteins in *G. lamblia*.** List of human cleavage and polyadenylation factors and putative *G. lamblia* orthologs. Human proteins were taken from (Tian & Graber, 2012). Orthologs for *G. lamblia* were identified as described in methods.
