# Supplementary figures and images for: Precise gene models using long-read sequencing reveal a unique poly(A) signal in Giardia lamblia
Source: RNA. 2022 May;28(5):668–82. doi: 10.1261/rna.078793.121 (PMC9014877; doi:10.1261/rna.078793.121)

A

|                  | # of genes | # of sites |
|------------------|------------|------------|
| QuantSeq rep 1   | 3716       | 4298       |
| QuantSeq rep 2   | 3738       | 4306       |
| QuantSeq overlap | 3558       | 4126       |
| ONT validation   | 2630       | 2764       |

B

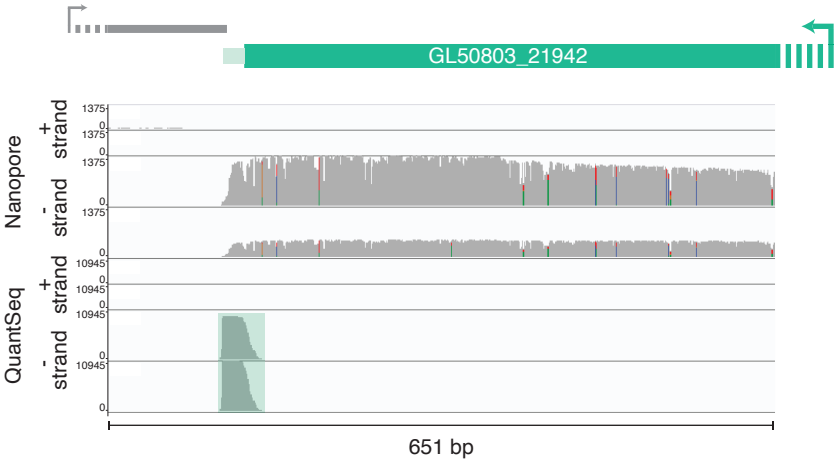

Supplement: Supplemental Material [file supp_078793.121_Supplemental_Figure_S1.pdf]

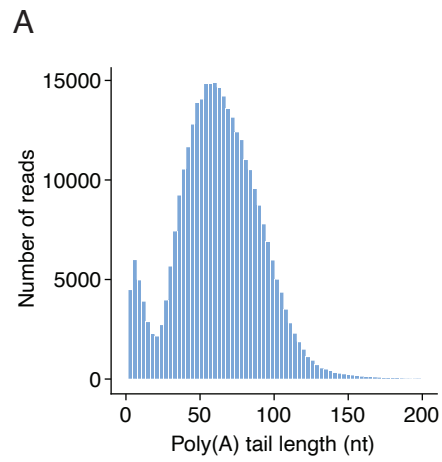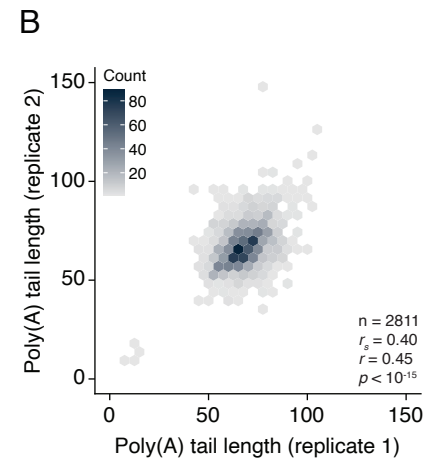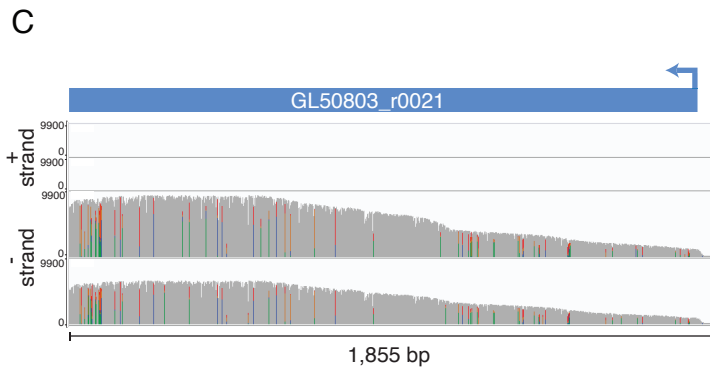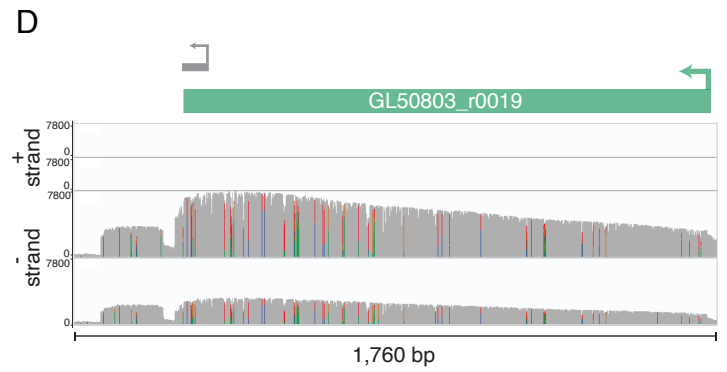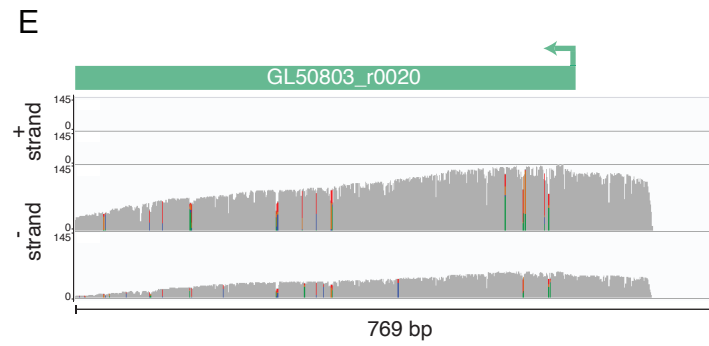

Supplement: Supplemental Material [file supp_078793.121_Supplemental_Figure_S2.pdf]

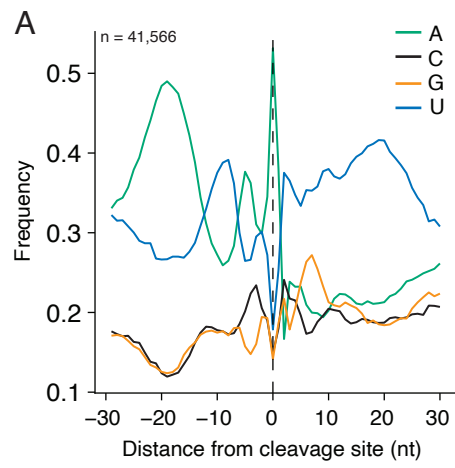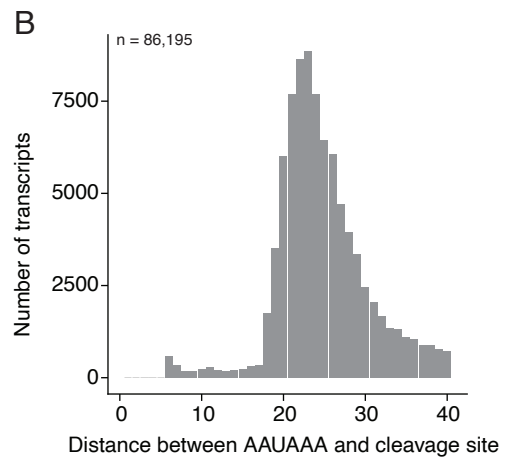

Supplement: Supplemental Material [file supp_078793.121_Supplemental_Figure_S3.pdf]

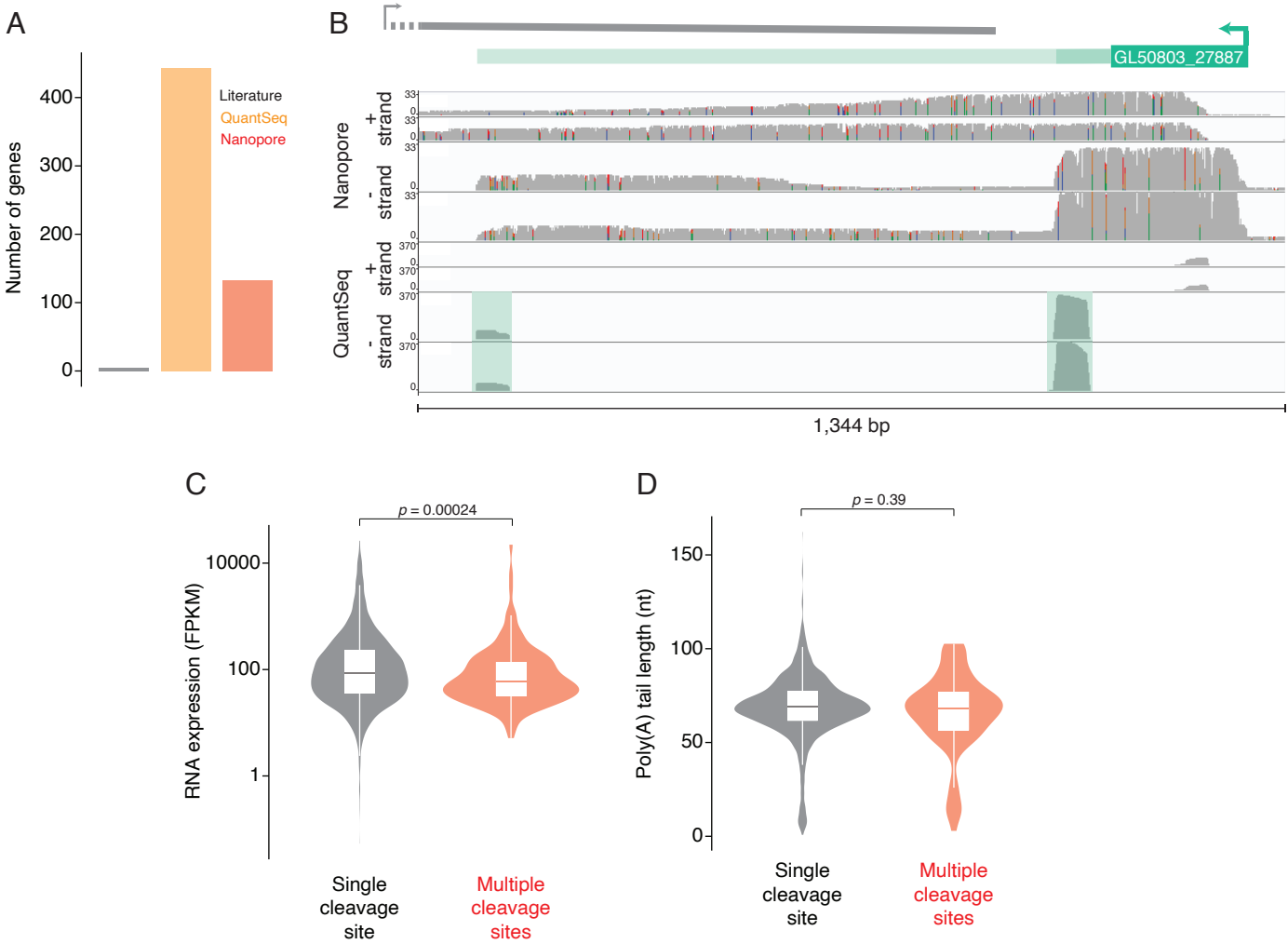

Supplement: Supplemental Material [file supp_078793.121_Supplemental_Figure_S4.pdf]
